# Contrasting effects of different molecular crowding environments on base‐pair opening/closing dynamics of DNA triplex structures

**DOI:** 10.1111/febs.70503

**Published:** 2026-03-25

**Authors:** Tomoki Sakamoto, Yudai Yamaoki, Takashi Nagata, Masato Katahira

**Affiliations:** ^1^ Institute of Advanced Energy Kyoto University Uji Kyoto Japan; ^2^ Graduate School of Energy Science Kyoto University Uji Kyoto Japan; ^3^ Department of Molecular Biophysics Tokyo University of Pharmacy and Life Sciences Tokyo Japan; ^4^ Integrated Research Center for Carbon Negative Science Kyoto University Uji Kyoto Japan; ^5^ Biomass Product Tree Industry−Academia Collaborative Research Laboratory Kyoto University Uji Kyoto Japan

**Keywords:** base pair opening/closing dynamics, molecular crowding, NMR, relaxation dispersion, triplex structure

## Abstract

DNA triplexes contribute to genomic instability and gene regulation. To understand how the molecular crowding environment (MCE) inside cells influences the dynamics of DNA triplex structures, we investigated base‐pair opening and closing (BPOC) dynamics using crowding agents that mimic intracellular MCE. We report, for the first time, quantitative rate constants for opening ($k_{\text{open}}$) and closing ($k_{\text{close}}$) of individual base pairs under MCE. While MCE preserved the overall triplex structure, it significantly altered the lifetimes of the closed ($\tau_{\text{closed}}=1/k_{\text{open}}$) and open ($\tau_{\text{open}}=1/k_{\text{close}}$) states, and the standard Gibbs energy difference (${\Delta G}_{\text{open}}^{○}=-\mathrm{RT}\ln K_{\text{open}}$, $K_{\text{open}}=\frac{k_{\text{open}}}{k_{\text{close}}}=\frac{\tau_{\text{open}}}{\tau_{\text{closed}}}$). Notably, we observed distinct changes in BPOC dynamics of the third strand. Ficoll PM 70, which enhances excluded‐volume effects, lengthened both $\tau_{\text{closed}}$ and $\tau_{\text{open}}$ across most base pairs, with a larger increase in $\tau_{\text{closed}}$, resulting in an increased ${\Delta G}_{\text{open}}^{○}$. In contrast, PEG 200, which reduces water activity, increased $\tau_{\text{open}}$ for most base pairs except for those near the helix center, while PEG 200 either decreased or increased $\tau_{\text{closed}}$ only slightly. As a result, the $\frac{\tau_{\text{open}}}{\tau_{\text{closed}}}$ ratio increased, resulting in a decreased ${\Delta G}_{\text{open}}^{○}$. These findings deepen mechanistic understanding of molecular‐crowding effects on DNA‐triplex stability and dynamics, providing new cellular regulatory perspectives.

## Graphical abstract

Base‐pair opening and closing regulate nucleic‐acid structure, stability, and function, but how these motions behave under intracellular molecular crowding remains unclear. Using NMR, we quantified these dynamics in a DNA triplex under two crowder‐reconstituted environments that mimic cellular crowding. The triplex structure remained intact, yet each environment produced distinct changes in base‐pair lifetimes and energetics, revealing how crowding modulates triplex stability.

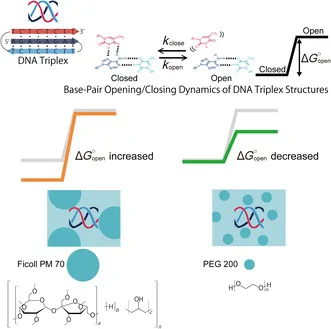

**Abbreviations**
BPOC: Base‐Pair Opening and Closing
Crowders: molecular crowding agents
HBPs: Hoogsteen Base Pairs
MCE: Molecular Crowding Environment
PT‐ODN: OligoDeoxyNucleotide that forms a Parallel Triplex structure
RD: Relaxation‐Dispersion
WCBPs: Watson–Crick Base Pairs
WMT: Water‐Magnetization‐Transfer

## Introduction

DNA, the fundamental carrier of genetic information in living organisms, is best known for its duplex structure [1, 2, 3]. However, DNA can also form noncanonical structures, including triplexes, which are becoming increasingly recognized as biologically significant [4, 5, 6]; such triplexes are involved in regulating gene expression [7, 8] and pausing replication [9], and contribute to genetic instability [10, 11, 12]. A DNA triplex forms when a triplex‐forming oligonucleotide (TFO) binds to the major groove of double‐stranded DNA. A duplex typically consists of a purine‐rich strand and a pyrimidine‐rich strand. A parallel triplex structure is formed when the TFO is aligned parallel to the purine‐rich strand of the duplex through Hoogsteen base pairs (HBPs).

The functions of nucleic acids are governed by both their structures and dynamics [13, 14, 15, 16, 17, 18, 19, 20, 21, 22, 23, 24, 25, 26, 27, 28]. In this regard, NMR spectroscopy is a powerful technique for probing nucleic acid dynamics at atomic resolution on the millisecond‐to‐submillisecond timescales. For example, conformational exchange between Watson–Crick base pairs (WCBPs) and mismatch‐like base pairs, which can be triggered by epitranscriptomic modifications such as N6‐methyladenosine (m^6^A), modulate RNA hybridization rates and, consequently, cellular response times [13]. Equilibria between WCBPs and HBPs in DNA duplexes [14, 15, 16, 17] play crucial roles in cellular stress responses and are vital for maintaining genomic stability [18]. For instance, oxidative stress can disrupt these equilibria [19] leading to DNA damage that is more challenging to repair [19]. Additionally, epigenetic DNA modifications impact base‐pair opening and closing (BPOC) dynamics and influence DNA–protein interactions as a consequence [20].

The cellular environment, often referred to as a ‘molecular crowding environment’ (MCE), is characterized by high concentrations of solutes such as proteins and nucleic acids that typically range between 50 and 400 g·L^−1^ [29, 30, 31, 32, 33, 34]. This crowding amplifies the excluded volume effect, reduces water activity, and increases nonspecific interactions, all of which significantly affect the structures and dynamics of nucleic acids and their interactions [33, 34].

Base‐pair opening reportedly regulates nucleic‐acid–protein interactions and plays a role in various biological processes [20, 35, 36]. We previously used in‐cell NMR techniques to demonstrate that living human cells exhibit higher base‐pair‐opening frequencies for RNA‐hairpin and DNA‐G‐quadruplex than under *in vitro* conditions [37, 38, 39, 40, 41, 42, 43]. However, the BPOC dynamics of DNA triplexes in cellular environments remain poorly understood. We previously discovered that analyzing DNA triplexes using in‐cell NMR spectroscopy requires more than 10 h of signal accumulation [43]. Hence, obtaining high‐quality in‐cell NMR signals for dynamic analysis is technically challenging given the potential for oligonucleotide degradation during such extended periods.

**Fig. 1:**
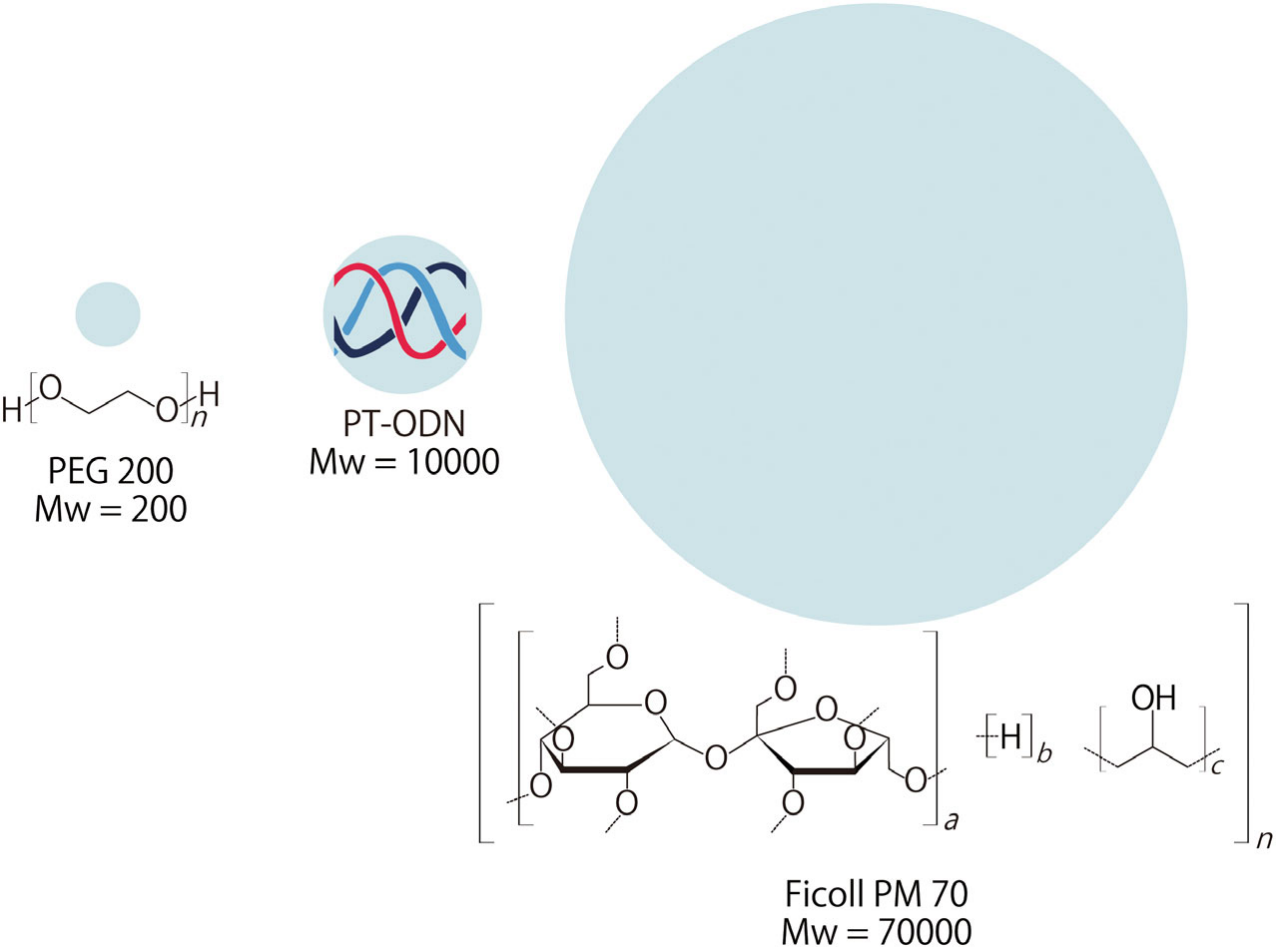
Schematic depicting crowder‐size differences. Light blue circles are scaled to the corresponding hydrodynamic radii and were generated assuming that PEG 200, Ficoll PM 70, and oligodeoxynucleotide that forms a parallel triplex structure (PT‐ODN) are spherical. The hydrodynamic radii of PEG 200, Ficoll PM 70, and PT‐ODN were set to 0.49 nm, [84] 4.7 nm, [85] and 1.2 nm, respectively. The hydrodynamic radius of PT‐ODN was estimated from the inter‐base‐pair distances in its B‐form double‐stranded DNA, which was calculated as: 0.34 × (8–1) × 1/2 = 1.2 nm).

In this study, we aimed to determine the opening and closing rate constants ($k_{\text{open}}$ and $k_{\text{close}}$) that govern BPOC dynamics in DNA triplexes under *in vitro* conditions using molecular crowding agents (crowders). We employed Ficoll PM 70 (MW 70000), a sucrose‐based polymer, and polyethylene glycol (PEG) 200 (MW 200) (Fig. 1). The use of such crowders *in vitro* enables mimicking of the crowded intracellular environment and facilitates the detailed analysis of BPOC dynamics under controlled conditions.

**Fig. 2:**
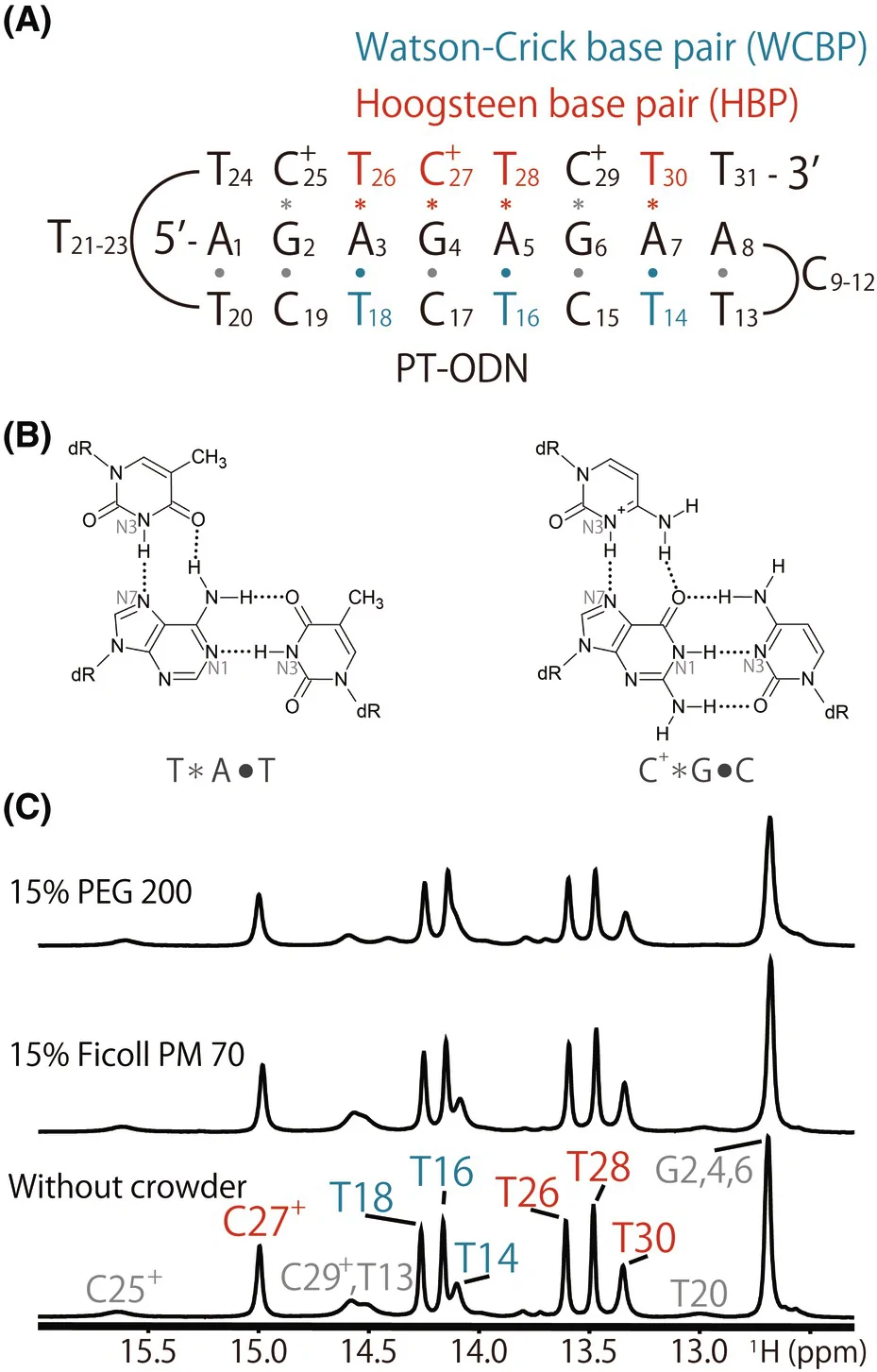
PT‐ODN and its 1D ^1^H NMR spectra in dilute and Molecular Crowding Environments (MCEs). (A) Secondary structure of the oligodeoxynucleotide that forms a parallel triplex structure (PT‐ODN). Residues whose imino proton signals were analyzed are highlighted: Watson–Crick base pairs (WCBPs) in sky blue and Hoogsteen base pairs (HBPs) in red, respectively. The NMR solution structure of a DNA triplex with a sequence closely related to that of PT‐ODN, in which the loop region is replaced with ethylene glycol linkers, has been determined (PDB: 1D3X), demonstrating that substituting the loop segment of the PT‐ODN sequence (C9–C12 and T21–T23) with ethylene glycol linkers does not significantly perturb the overall triplex structure [44, 65]. (B) Chemical structures of the base triples T*A**•**T and C^+^ *G**•**C present in PT‐ODN. (C) 1D ^1^H NMR spectra of the imino proton region of PT‐ODN under three conditions: dilute (bottom), 15% Ficoll PM 70 (middle), and 15% PEG 200 (top). Samples contained 1.5 mm PT‐ODN in 20 mm sodium phosphate buffer (pH 7.0; 8 mm NaH_2_PO_4_, 12 mm Na_2_HPO_4_) supplemented with 168 mm NaCl and 5% D_2_O. Spectra were acquired once per condition (*n* = 1 per condition). Spectra were recorded at 25 °C with 64 scans.

We used an oligodeoxynucleotide that forms a parallel triplex structure (PT‐ODN) (Fig. 2A,B) [44]. Our previous studies revealed that this PT‐ODN forms a similar structure at pH 7.0 in both *in vitro* [44] and cellular environments [43]. Previous investigations into the effects of MCE on DNA triplexes under *in vitro* conditions have primarily relied on UV spectroscopy [45, 46, 47, 48, 49, 50, 51]; these studies typically assessed the impact of MCE by measuring the melting temperature (*T*_m_) of the triplex. Research by Spink *et al*. showed that MCE effects result from a combination of excluded‐volume effects and reduced water activity [45, 46]. While the excluded‐volume effect and low water activity have been shown to contribute to DNA‐triplex stabilization and destabilization, respectively [45, 46], how MCE affects individual base pairs within a DNA triplex structure remains unclear.

Studies of BPOC dynamics by NMR spectroscopy have conventionally relied on imino–proton exchange experiments using the water‐magnetization‐transfer (WMT) method under varying concentrations of a base catalyst [52, 53, 54] (The detailed theoretical framework for BPOC dynamics is described in the section ‘Theoretical framework for analyzing base‐pair opening/closing (BPOC) dynamics’). Such measurements are typically performed under mildly alkaline conditions (e.g., pH 8.0) using tris(hydroxymethyl)aminomethane (Tris) as the base catalyst [52, 53, 54]. However, parallel DNA triplexes contain C^+^ *G HBPs (Fig. 2B right) stabilized by protonated cytosine, whose imino protons are undetectable at pH 8.0, precluding analysis of these HBPs. Lowering the pH to 7.0 preserves C^+^ *G HBPs but markedly reduces the concentration of the base form of Tris, (HOCH_2_)_3_CNH_2_, thereby decreasing both the imino‐proton exchange rate and its dependence on catalyst concentration, which hampers determination of $k_{\text{open}}$ and $k_{\text{close}}$. Substituting sodium phosphate buffer, whose second $pK_{a}$ (7.21) is closer to pH 7.0, increases the concentration of the base form (HPO_4_^2−^) relative to Tris. Nevertheless, because its basicity is lower, the probability of proton transfer to the imino group is reduced, again yielding small changes in the exchange rate with catalyst concentration. As a result, the exchange rate of the imino proton and its dependence on catalyst concentration remain small, making accurate determination of $k_{\text{open}}$ and $k_{\text{close}}$ difficult. Increasing the base‐catalyst concentration could in principle enhance the exchange rate and its dependence on catalyst concentration, but such high concentrations have been reported to alter DNA dynamics [52, 53]. Thus, it is difficult to determine BPOC dynamics at pH 7.0 solely by imino–proton exchange measurements.

To overcome this limitation, we developed a combined WMT/^1^H *R*₁ρ relaxation‐dispersion (RD) approach that enables determination of BPOC dynamics at pH 7.0 without varying the base‐catalyst concentration. In this method, only a single catalyst concentration is required, and the need to monitor exchange‐rate changes as a function of catalyst concentration is eliminated. This allows BPOC dynamics of DNA triplexes to be probed under physiologically relevant pH 7.0 conditions, minimizing pH‐induced structural perturbations [11, 55]. The approach is broadly applicable to other nucleic‐acid structures, including duplexes and quadruplexes, and allows BPOC dynamics to be assessed at pH 7.0 while avoiding pH‐induced perturbations to the structural stability. It is particularly advantageous for characterizing BPOC dynamics under MCEs while avoiding artifacts associated with high catalyst concentrations.

Herein, we show that while the overall structure of the DNA triplex remains unchanged under MCEs, its BPOC dynamics are affected by the MCE. We observed the following changes in BPOC dynamics by focusing on the third HBP strand: The addition of Ficoll PM 70, which enhances the excluded‐volume effect prolonged the lifetimes of both the closed and open states of most base pairs, with the closed state notably more‐significantly prolonged, resulting in a larger standard Gibbs energy change between the closed and open states, indicative of base‐pair stabilization. In contrast, the addition of PEG 200, which reduces water activity, lengthened the lifetime of the open state for most base pairs except for those near the helix center, while the addition of PEG 200 either shortened or lengthened the lifetime of the closed state only slightly. As a result, the $\frac{\tau_{\text{open}}}{\tau_{\text{closed}}}$ ratio increased, resulting in a decreased ${\Delta G}_{\text{open}}^{○}$. These findings highlight how different MCEs distinctly influence the BPOC dynamics of DNA triplexes. Given that intracellular crowding varies during processes such as the cell cycle and senescence, DNA‐triplex dynamics may plausibly change in response to such environmental changes, which potentially affects essential cellular functions.

## Results

### DNA triplex structures in molecular crowding environments (MCEs)

In this section, we investigated the effects of two crowders with distinct molecular weights on the structure of a DNA triplex. We employed Ficoll PM 70, a sucrose‐based polymer with a molecular weight of 70 000, and PEG 200, with a molecular weight of 200 (Fig. 1). One‐dimensional (1D) ^1^H NMR spectra of PT‐ODN were recorded at 25 °C in the absence of crowders (Fig. 2C, bottom) and in the presence of 15% Ficoll PM 70 (Fig. 2C, middle) or 15% PEG 200 (Fig. 2C, top). These spectra revealed imino proton signals corresponding to the ^1^H^N1^ of guanine and the ^1^H^N3^ of thymine and cytosine residues. Based on previous studies [43, 44], we assigned these signals to G2, G4, G6, T13, T14, T16, T18, and T20 in the WCBPs, and to C25^+^, T26, C27^+^, T28, C29^+^, and T30 in the HBPs (Fig. 2C, bottom). No signals were observed for the imino protons of T24 and T31. Base pairs with detectable imino signals are indicated in the schematic representation of PT‐ODN (Fig. 2A), where dots (•) denote WCBPs and asterisks (*) represent HBPs. Among these, the well‐resolved signals analyzed in this study are further highlighted in sky blue (WCBPs) and red (HBPs).

In the absence of a crowder, the imino proton signals of PT‐ODN could be classified into three groups (Fig. 2C, bottom). The first group consisted of sharp and well‐resolved signals corresponding to T14, T16, T18, T26, C27^+^, T28, and T30. The second group included broader signals from T13, T20, C25^+^, and C29^+^, whereas the third group comprised overlapping signals corresponding to G2, G4, and G6. The signal broadening observed for T13, T20, C25^+^, and C29^+^ is likely ascribable to end fraying due to their proximity to the ends of the secondary structure. For subsequent analyses, we focused primarily on the well‐resolved signals in the first group.

The addition of Ficoll PM 70 did not significantly affect the imino proton signals of T14, T16, T18, T26, C27^+^, T28, and T30 (Fig. 2C, middle). Similarly, PEG 200 had little influence on these signals, except for a slight change observed for the T14 signal (Fig. 2C, top). These results indicate that the overall triplex structure of PT‐ODN remains largely unperturbed in the presence of either Ficoll PM 70 or PEG 200, suggesting that these MCEs do not induce major structural alterations.

### Theoretical framework for analyzing base‐pair opening/closing (BPOC) dynamics

The $k_{\mathrm{ex},1H}$, derived from WMT experiments, reflects the base‐catalyzed proton exchange between imino and bulk‐water protons. This process is modeled using a two‐state system, following the theoretical framework established by Guéron and Leroy and further developed by Stivers and co‐workers. In the closed state, the imino proton is hydrogen‐bonded and inaccessible to solvent, whereas in the open state, disruption of this hydrogen bond allows exchange with water protons (Eqn 1) [36, 52, 54, 56]:
(1)

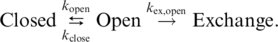




Previously, Powell *et al*. investigated the individual exchange behavior of A•T and G•C WCBPs, as well as T*A and C^+^ *G HBPs of PT‐ODN under dilute *in vitro* conditions [57], which revealed that these base pairs open independently and that the open state is transient and thermodynamically less stable than the closed state, with $k_{\text{close}}$ >> $k_{\text{open}}$. $k_{\mathrm{ex},1H}$ can be determined using Eqn 2 [57]:

(2) $$k_{\mathrm{ex},1H}=\frac{k_{\text{open}}k_{\mathrm{ex},\text{open}}}{k_{\text{close}}+k_{\mathrm{ex},\text{open}}}.$$

The equilibrium constant for base‐pair opening is defined as $K_{\text{open}}=k_{\text{open}}/k_{\text{close}}$, while the change in the standard Gibbs energy difference between the closed and open states of the base pairs is given by ${\Delta G}_{\text{open}}^{○}=-\mathrm{RT}\ln K_{\text{open}}$. The lifetimes of the closed and open states ($\tau_{\text{open}}$ and $\tau_{\text{closed}}$) are defined as $1/k_{\text{close}}$ and $1/k_{\text{open}}$, respectively.

The imino‐proton exchange rate in the open state ($k_{\mathrm{ex},\text{open}}$) is influenced by two proton‐acceptor types [52, 57]. The first is the ${\mathrm{HPO}}_{4}^{2-}$ in the solvent, and is referred to as the ‘external proton acceptor’ in this study [58]. The second includes the N1 atoms of the adenine moieties in the A•T WCBPs, the N7 atoms of the adenine moieties in the T*A Hoogsteen base pairs (HBPs), the N3 atoms of the thymine moieties in the G•C WCBPs, and the N7 atoms of the guanine moieties in the C^+^ *G HBPs, which are referred to as ‘internal proton acceptors’. These two catalysts, an external proton acceptor and internal proton acceptors, promote proton exchange in the open state [52]. Accordingly, the overall proton exchange rate in the open state is the sum of the exchange rates involving both the external and internal acceptors ($k_{\mathrm{ex},\text{open}}^{\mathrm{ext}}$ and $k_{\mathrm{ex},\text{open}}^{\mathrm{int}}$, respectively): [57].

(3) $$k_{\mathrm{ex},\text{open}}=k_{\mathrm{ex},\text{open}}^{\mathrm{ext}}+k_{\mathrm{ex},\text{open}}^{\mathrm{int}}$$

$k_{\mathrm{ex},\text{open}}^{\mathrm{ext}}$ is directly proportional to the concentration of the acceptor: [52].

(4) $$k_{\mathrm{ex},\text{open}}^{\mathrm{ext}}=k_{A}\left[\text{acceptor}\right]$$

Rate constant $k_{A}$ depends on the pK values of the imino group and the acceptor, as well as the collision frequency ($k_{\text{coll}}$):

(5) $$k_{A}=k_{\text{coll}}F$$

where fraction F represents the probability of proton transfer within a transient hydrogen‐bonded complex, and is given by:

(6) $$F={\left(1+10^{-\mathrm{\Delta p}K}\right)}^{-1}$$

(7) $$\mathrm{\Delta p}K=pK\left(\text{acceptor}\right)-pK\left(\mathrm{NH}\right)$$

The rate constant $k_{A}$ is defined as $k_{A}=\alpha k_{A}^{0}$, where $k_{A}^{0}$ is the proton exchange rate of the free nucleoside, and α is an empirical constant quantifying the relative accessibility of the imino group in the base pair relative to that in the free nucleoside. Experimental studies indicate that α is typically close to 1 [56].

The values of $k_{\text{open}}$ and $k_{\text{close}}$ were determined by simultaneously solving Eqns 2,14 (Eqn 14 is provided in the Material and Methods section), after substituting the experimentally determined $k_{\mathrm{ex}}$ and $k_{\mathrm{ex},1H}$ values together with $k_{\mathrm{ex},\text{open}}$ value calculated using data from the literature and experimentally (calculational details are provided in the next section).

### Calculating the exchange rate between imino and water protons in the open state (*k*_ex,open_)

This section describes how the exchange rates between imino and water protons in the open state ($k_{\mathrm{ex},\text{open}}$) are calculated for the various base‐pair types in PT‐ODN, following the theoretical framework outlined in the previous section; it describes the derivation of $k_{\mathrm{ex},\text{open}}$ for A•T WCBPs, T*A HBPs, and protonated C^+^ *G HBPs.

#### A•T WCBPs

The external exchange rate ($k_{\mathrm{ex},\text{open}}^{\mathrm{ext}}$) for A•T WCBPs was determined as follows.

The phosphate buffer concentration was set to 20 mm at pH 7.0 and the concentration of the specific proton acceptor species ($\left[{\mathrm{HPO}}_{4}^{2-}\right]$) was calculated using the Equation: [59, 60].

$$\left[{\mathrm{HPO}}_{4}^{2-}\right]=\left[{H_{2}\mathrm{PO}}_{4}^{-}\right]/\left(1+10^{pK_{a2}-\mathrm{pH}}\right)$$

$\left[{\mathrm{HPO}}_{4}^{2-}\right]$ was calculated to be 7.63 mm given that ${pK}_{a2}$ = 7.21 for the second dissociation of phosphate and the buffer pH is 7.0. The collision frequency ($k_{\text{coll}}$) was determined to be 5.98 × 10^8^ s^−1^ according to a previous study; [58] this value was calculated from the $k_{\mathrm{TR},\mathrm{ext}}$ (which corresponds to our $k_{A}^{0}$) value of dTTP in 1 mm phosphate (pH 6.8), which corresponds to the conditions used in that study [58]. The *F* value, which is the proton‐transfer probability, was calculated using the p*K* values of ${\mathrm{HPO}}_{4}^{2-}$ and the N3 position of thymine (7.21 and 9.94, respectively); hence ΔpK = −2.73, resulting in an F value of 1.86 × 10^−3^.

The $k_{\mathrm{ex},\text{open}}^{\mathrm{ext}}$ value was calculated to be 8.49 × 10^3^ s^−1^ by applying $\left[{\mathrm{HPO}}_{4}^{2-}\right]=7.63\times 10^{-3}$, $k_{\text{coll}}=$5.98 × 10^8^, F= 1.86 × 10^−3^, and *α* = 1 to Eqs. 4 and 5, The internal exchange rate ($k_{\mathrm{ex},\text{open}}^{\mathrm{int}}$) has previously been established to be 3 × 10^4^ s^−1^ [52, 57].

The overall exchange rate for A•T WCBPs ($k_{\mathrm{ex},\text{open}}$) was finally calculated to be 3.85 × 10^4^ s^−1^ using Eqn 3.

#### T*A HBPs

The external exchange rate ($k_{\mathrm{ex},\text{open}}^{\mathrm{ext}}$) for the T*A HBPs was determined using the same approach as that used for the A•T WCBPs. The $k_{\mathrm{ex},\text{open}}^{\mathrm{ext}}$ value for the T*A HBPs is identical to that for the A•T WCBPs (i.e., 8.49 × 10^3^ s^−1^) using the same values for $\left[{\mathrm{HPO}}_{4}^{2-}\right]$, $k_{\text{coll}}$, F, and *α*.

The $k_{\mathrm{ex},\text{open}}^{\mathrm{int}}$ value for the T*A HBPs was derived based on the differences in the *F* value of the imino group and its acceptor; [52, 57] these *F* values were calculated for both the A•T WCBPs and T*A HBPs [52, 57]. This method is consistent with previous studies that characterized imino proton exchange in PT‐ODN [57]. ΔpK = −5.64 for the A•T WCBPs (pK = 4.3 for the N1 position of adenine and 9.94 for the N3 position of thymine) [61]. ΔpK = −8.94 for the T*A HBPs (assuming pK < 1 for the N7 position of adenine and 9.94 for the N3 position of thymine) [61]. Consequently, *F* values of 2.29 × 10^−6^ and 1.15 × 10^−9^ were calculated for the A•T WCBPs and T*A HBPs.

The $k_{\mathrm{ex},\text{open}}^{\mathrm{int}}$ value for the T*A HBPs was determined to be 15.1 s^−1^ by applying a ratio of 5.02 × 10^−4^ (i.e., 1.15 × 10^−9^/2.29 × 10^−6^) to the $k_{\mathrm{ex},\text{open}}^{\mathrm{int}}$ value for the A•T WCBPs (i.e., 3 × 10^4^ s^−1^; see A•T WCBPs subsection, above).

The overall $k_{\mathrm{ex},\text{open}}$ for the T*A HBPs was finally calculated to be 8.51 × 10^3^ s^−1^ using Eqn 3.

#### C^+^*G HBPs

The $k_{\text{coll}}$ value required to calculate the $k_{\mathrm{ex},\text{open}}^{\mathrm{ext}}$ value for the C^+^ *G HBPs was taken to be 5.98 × 10^8^ s^−1^ based on dTTP (see A•T WCBPs subsection, above), along with $\left[{\mathrm{HPO}}_{4}^{2-}\right]=$ 7.63 mm and *F* = 1, based on ΔpK = 2.61 (where pK = 4.6 for N3‐protonated cytosine and 7.21 for the ${\mathrm{HPO}}_{4}^{2-}$ acceptor). Hence, the $k_{\mathrm{ex},\text{open}}^{\mathrm{ext}}$ value was calculated to be 4.56 × 10^6^ s^−1^ when *α* = 1.

The $k_{\mathrm{ex},\text{open}}^{\mathrm{int}}$ value was calculated using the same method described in the T*A HBPs subsection (above). ΔpK = −4.6 for the G•C WCBPs (pK = 9.2 for the N1 position of guanine and 4.6 for the N3 position of cytosine) [61], while ΔpK = −1.3 for the C^+^ *G HBPs (pK = 3.3 for the N7 position of guanine and 4.6 for the N3 position of cytosine) [61]. F values of 2.51 × 10^−5^ and 4.77 × 10^2^ were calculated for the G•C WCBPs and C^+^ *G HBPs respectively.

The $k_{\mathrm{ex},\text{open}}^{\mathrm{int}}$ value for the C^+^ *G HBPs was determined to be 1.9 × 10^9^ s^−1^ by applying a ratio of 1.9 × 10^3^ (i.e., 4.77 × 10^−2^/2.51 × 10^−5^) to the $k_{\mathrm{ex},\text{open}}^{\mathrm{int}}$ value of 1 × 10^6^ for the G•C WCBPs.

The overall $k_{\mathrm{ex},\text{open}}$ for the C^+^*G HBPs was finally calculated to be 1.90 × 10^9^ s^−1^ using Eqn 3.

### Exchange rates between the closed and open states and water–imino proton‐exchange rate of PT‐ODN in the absence and presence of crowders

**Fig. 3:**
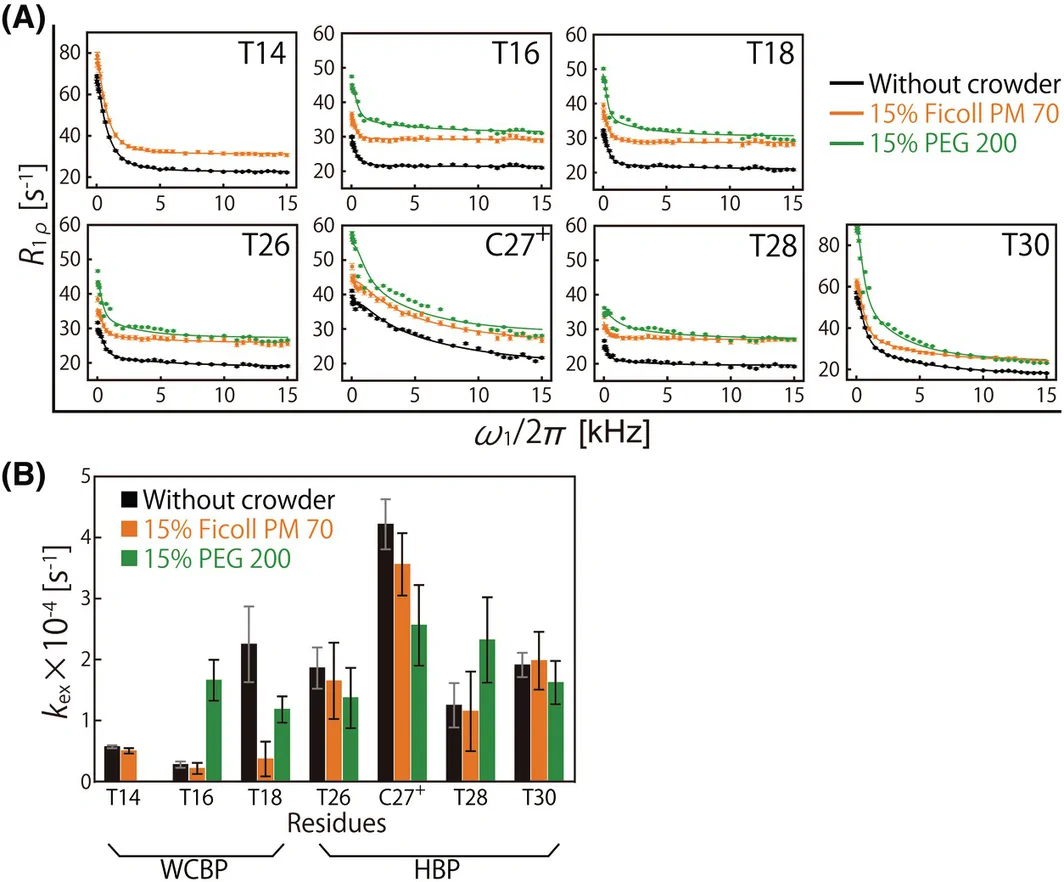
^1^H *R*_1ρ_ Relaxation‐Dispersion (RD) experiments involving oligodeoxynucleotide that forms a parallel triplex structure (PT‐ODN). (A) RD profiles for individual residues: Watson–Crick base pairs (WCBPs), upper panels; Hoogsteen base pairs (HBPs) lower panels. Profiles are shown under different conditions: without crowder (black), with 15% Ficoll (orange), and with 15% PEG 200 (green). (B) $k_{\mathrm{ex}}$ values for each residue under the following conditions. RD measurements were performed using 1.5 mm PT‐ODN in 20 mm sodium phosphate buffer (pH 7.0; 8 mm NaH_2_PO_4_, 12 mm Na_2_HPO_4_), with 168 mm NaCl and 5% D_2_O. Data were acquired once per condition (*n* = 1 per condition). NMR measurements were performed at 25 °C with 36 scans. In the presence of PEG 200, the T14 imino‐proton signal overlapped with another resonance (Fig. 2C, top), precluding reliable analysis. The error bars for each point in panel (A) represent the expected uncertainty in the signal intensity estimated from the noise level measured in signal‐free regions of the NMR spectra. Assuming that the $R_{1\rho }$ values are normally distributed within this uncertainty, 2000 synthetic intensity datasets were generated. Each synthetic dataset was fitted independently, and in panel (B) the uncertainties derived from the resulting distribution of fitted parameters are shown as error bars on the best‐fit values obtained from the experimental (non‐synthetic) data.

We investigated the influence of crowders on the kinetics of BPOC dynamics in PT‐ODN by examining their effects on the exchange rate between the closed and open states ($k_{\mathrm{ex}}$) of selected imino protons. Specifically, we analyzed T14, T16, and T18 in the WCBPs, as well as T26, C27^+^, T28, and T30 in the HBPs. The $R_{1\rho }$ value for each imino proton was calculated using Eqn 8 and plotted as a function of spin‐lock pulse strength (Fig. 3A). The resulting RD decay profiles indicated the presence of at least two exchange states for the base pairs examined in this study. The $k_{\mathrm{ex}}$ values were extracted by fitting these profiles to a two‐state exchange model described by Eqn 9, assuming that the exchanging states correspond to the closed and open conformers (Fig. 3B and Tables 1, 2, 3). The $k_{\mathrm{ex}}$ rate constant reflects the speed at which equilibrium is re‐established after perturbation [21]. Other parameters obtained from the fitting ($R_{2}^{0}$ and $p_{A}p_{B}\Delta \omega^{2}$) are listed in Tables 4, 5, 6.

**Table 1:** Values of $k_{\mathrm{ex}}$, $k_{\mathrm{ex},1H}$, $k_{\text{open}}$, $k_{\text{close}}$, $\tau_{\text{closed}}$, $\tau_{\text{open}}$, and ${\Delta G}_{\text{open}}^{○}$ in the absence of crowders.

	$k_{\mathrm{ex}}$×10^−4^ [s^−1^]	$k_{\mathrm{ex},1H}$ [s^−1^]	$k_{\text{open}}$ [s^−1^]	$k_{\text{close}}$ ×10^−4^ [s^−1^]	$\tau_{\text{closed}}$ [s]	$\tau_{\text{open}}$ [ms]	${\Delta G}_{\text{open}}^{○}$ [kJ]
T14	0.57 ± 0.02	1.37 ± 0.03	1.57 ± 0.04	0.57 ± 0.02	0.64 ± 0.014	0.18 ± 0.0074	20 ± 0.1
T16	0.28 ± 0.05	0.53 ± 0.02	0.59 ± 0.02	0.28 ± 0.05	1.8 ± 0.070	0.36 ± 0.066	21 ± 0.5
T18	2.25 ± 0.62	1.05 ± 0.02	1.66 ± 0.17	2.25 ± 0.62	0.60 ± 0.062	0.044 ± 0.012	24 ± 0.7
T26	1.86 ± 0.34	3.42 ± 0.02	10.9 ± 0.30	1.86 ± 0.34	0.092 ± 0.011	0.054 ± 0.0098	18 ± 0.5
C27^+^	4.22 ± 0.41	15.3 ± 1.49	15.3 ± 3.53	4.22 ± 0.41	0.065 ± 0.0063	0.024 ± 0.0023	20 ± 0.6
T28	1.25 ± 0.36	3.45 ± 0.02	8.51 ± 0.33	1.25 ± 0.36	0.12 ± 0.020	0.080 ± 0.023	18 ± 0.7
T30	1.91 ± 0.20	10.1 ± 0.05	32.6 ± 0.53	1.90 ± 0.20	0.031 ± 0.0022	0.053 ± 0.0055	16 ± 0.3

**Table 2:** Values of $k_{\mathrm{ex}}$, $k_{\mathrm{ex},1H}$, $k_{\text{open}}$, $k_{\text{close}}$, $\tau_{\text{closed}}$, $\tau_{\text{open}}$, and ${\Delta G}_{\text{open}}^{○}$ in the presence of 15% Ficoll PM 70.

	$k_{\mathrm{ex}}$×10^−4^ [s^−1^]	$k_{\mathrm{ex},1H}$ [s^−1^]	$k_{\text{open}}$ [s^−1^]	$k_{\text{close}}$ ×10^−4^ [s^−1^]	$\tau_{\text{closed}}$ [s]	$\tau_{\text{open}}$ [ms]	${\Delta G}_{\text{open}}^{○}$ [kJ]
T14	0.50 ± 0.04	1.24 ± 0.09	1.40 ± 0.10	0.50 ± 0.04	0.71 ± 0.052	0.20 ± 0.017	20 ± 0.3
T16	0.21 ± 0.09	0.46 ± 0.05	0.49 ± 0.05	0.21 ± 0.09	2.1 ± 0.23	0.47 ± 0.20	21 ± 1.1
T18	0.37 ± 0.28	0.76 ± 0.06	0.83 ± 0.09	0.37 ± 0.28	1.2 ± 0.12	0.27 ± 0.21	21 ± 1.9
T26	1.65 ± 0.63	2.27 ± 0.06	6.66 ± 1.68	1.65 ± 0.63	0.15 ± 0.038	0.061 ± 0.023	19 ± 1.1
C27^+^	3.57 ± 0.51	13.0 ± 1.26	13.0 ± 1.26	3.57 ± 0.51	0.077 ± 0.0075	0.028 ± 0.0041	20 ± 0.4
T28	1.15 ± 0.65	2.31 ± 0.05	5.42 ± 1.77	1.15 ± 0.65	0.18 ± 0.060	0.087 ± 0.049	19 ± 1.6
T30	1.98 ± 0.47	7.46 ± 0.38	24.8 ± 4.34	1.98 ± 0.47	0.040 ± 0.0070	0.050 ± 0.012	17 ± 0.7

**Table 3:** Values of $k_{\mathrm{ex}}$, $k_{\mathrm{ex},1H}$, $k_{\text{open}}$, $k_{\text{close}}$, $\tau_{\text{closed}}$, $\tau_{\text{open}}$, and ${\Delta G}_{\text{open}}^{○}$ in the presence of 15% PEG 200.

	$k_{\mathrm{ex}}$×10^−4^ [s^−1^]	$k_{\mathrm{ex},1H}$ [s^−1^]	$k_{\text{open}}$ [s^−1^]	$k_{\text{close}}$ ×10^−4^ [s^−1^]	$\tau_{\text{closed}}$ [s]	$\tau_{\text{open}}$ [ms]	${\Delta G}_{\text{open}}^{○}$ [kJ]
T16	1.66 ± 0.34	1.10 ± 0.03	1.57 ± 0.11	1.66 ± 0.34	0.64 ± 0.042	0.060 ± 0.012	23 ± 0.5
T18	1.18 ± 0.22	1.97 ± 0.09	2.57 ± 0.16	1.18 ± 0.22	0.39 ± 0.024	0.085 ± 0.016	21 ± 0.5
T26	1.37 ± 0.49	4.29 ± 0.04	11.2 ± 2.48	1.37 ± 0.49	0.089 ± 0.02	0.073 ± 0.026	18 ± 1.0
C27^+^	2.56 ± 0.66	14.1 ± 0.04	14.1 ± 0.04	2.56 ± 0.66	0.071 ± 0.0002	0.039 ± 0.01	19 ± 0.6
T28	2.32 ± 0.70	4.34 ± 0.03	16.1 ± 3.57	2.31 ± 0.70	0.062 ± 0.014	0.043 ± 0.013	18 ± 0.9
T30	1.62 ± 0.36	15.8 ± 1.29	46.0 ± 7.60	1.62 ± 0.36	0.022 ± 0.0036	0.062 ± 0.014	15 ± 0.7

**Table 4:** Values of $R_{2}$ and $p_{A}p_{B}\Delta \omega^{2}$ in the absence of crowders.

	$R_{2}^{0}$ [s^−1^]	$p_{A}p_{B}\Delta \omega^{2}$ [s^−2^]
T14	22.6 ± 0.1	2.43 × 10^5^ ± 1.1 × 10^4^
T16	21.4 ± 0.1	2.25 × 10^4^ ± 5.0 × 10^3^
T18	20.7 ± 0.2	2.28 × 10^5^ ± 6.3 × 10^4^
T26	18.8 ± 0.2	2.04 × 10^5^ ± 6.6 × 10^4^
C27^+^	18.8 ± 0.8	8.16 × 10^5^ ± 1.1 × 10^5^
T28	19.4 ± 0.2	8.32 × 10^4^ ± 8.2 × 10^4^
T30	17.2 ± 0.6	6.85 × 10^5^ ± 8.7 × 10^4^

**Table 5:** Values of $R_{2}$ and $p_{A}p_{B}\Delta \omega^{2}$ in the presence of 15% Ficoll PM 70.

	$R_{2}$ [s^−1^]	$p_{A}p_{B}\Delta \omega^{2}$ [s^−1^]
T14	31.1 ± 0.3	2.25 × 10^5^ ± 3.1 × 10^4^
T16	29.3 ± 0.2	1.41 × 10^4^ ± 5.9 × 10^3^
T18	28.7 ± 0.3	3.49 × 10^4^ ± 2.7 × 10^4^
T26	25.7 ± 0.7	2.39 × 10^5^ ± 3.4 × 10^6^
C27^+^	25.4 ± 0.9	6.98 × 10^5^ ± 5.7 × 10^5^
T28	26.9 ± 0.3	9.15 × 10^4^ ± 7.1 × 10^5^
T30	23.0 ± 0.9	7.20 × 10^5^ ± 1.8 × 10^5^

**Table 6:** Values of $R_{2}$ and $p_{A}p_{B}\Delta \omega^{2}$ in the presence of 15% PEG 200.

	$R_{2}$ [s^−1^]	$p_{A}p_{B}\Delta \omega^{2}$ [s^−1^]
T16	31.2 ± 0.3	2.26 × 10^5^ ± 7.9 × 10^4^
T18	30.4 ± 0.3	2.04 × 10^5^ ± 1.0 × 10^5^
T26	26.9 ± 0.6	2.36 × 10^5^ ± 1.7 × 10^5^
C27^+^	28.0 ± 1.8	7.78 × 10^5^ ± 8.3 × 10^5^
T28	26.9 ± 0.6	2.97 × 10^5^ ± 6.3 × 10^5^
T30	22.9 ± 2.0	9.99 × 10^5^ ± 2.9 × 10^5^

**Fig. 4:**
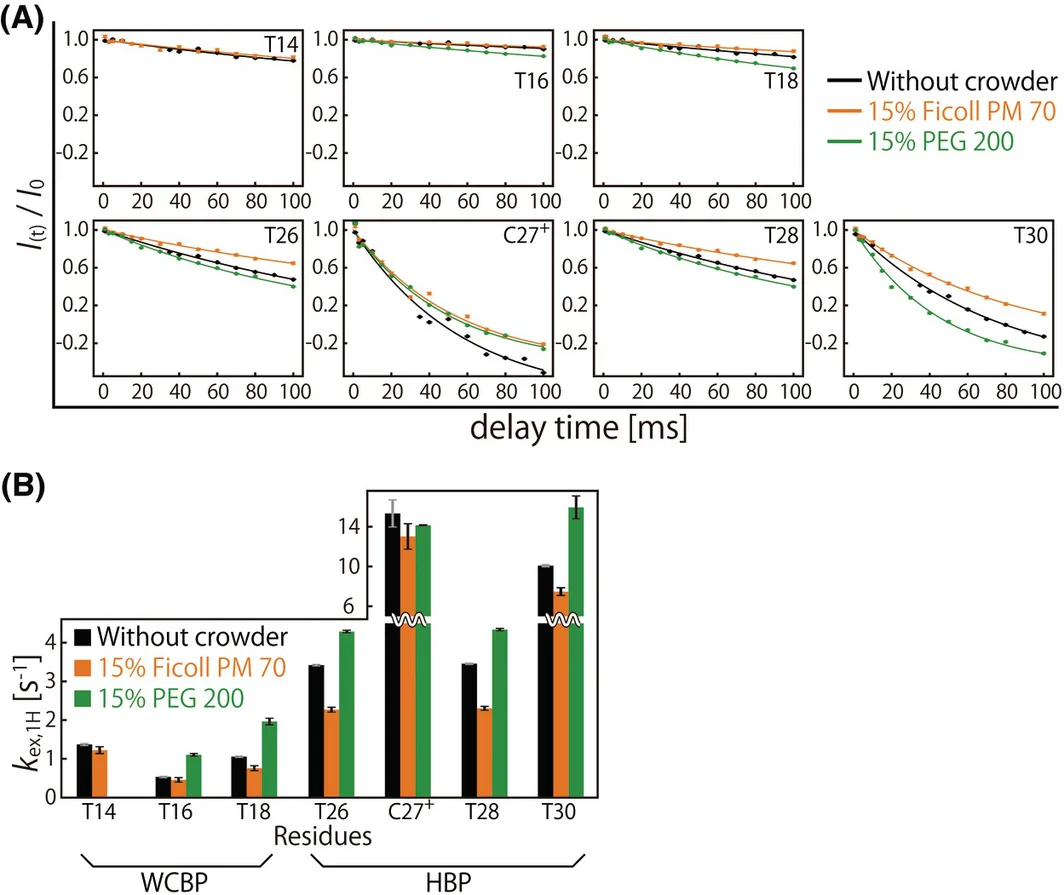
Water‐Magnetization‐Transfer (WMT) experiments involving oligodeoxynucleotide that forms a parallel triplex structure (PT‐ODN). (A) WMT data for individual residues: Watson–Crick base pairs (WCBPs), upper panels; Hoogsteen base pairs (HBPs), lower panels. The data are color‐coded based on experimental conditions: without crowder (black), with 15% Ficoll (orange), and with 15% PEG 200 (green). (B) $k_{\mathrm{ex},1H}$ values for each residue. WMT measurements were performed using 1.5 mm PT‐ODN in 20 mm sodium phosphate buffer (pH 7.0; 8 mm NaH_2_PO_4_, 12 mm Na_2_HPO_4_), with 168 mm NaCl and 5% D_2_O. Data were acquired once per condition (*n* = 1 per condition). NMR measurements were performed at 25 °C with 8 scans. In the presence of PEG 200, the T14 imino‐proton signal overlapped with another resonance (Fig. 2C, top), precluding reliable analysis. The error bars for each point in panel (A) represent the expected uncertainty in the signal intensity estimated from the noise level measured in signal‐free regions of the NMR spectra. Assuming that the $k_{\mathrm{ex},1H}$ values are normally distributed within this uncertainty, 500 synthetic intensity datasets were generated. Each synthetic dataset was fitted independently, and in panel (B) the uncertainties derived from the resulting distribution of fitted parameters are shown as error bars on the best‐fit values obtained from the experimental (non‐synthetic) data.

We investigated how MCE affects the water–imino proton exchange rate ($k_{\mathrm{ex},1H}$) in PT‐ODN by determining $k_{\mathrm{ex},1H}$ values for the imino protons of the T14, T16, and T18 in the WCBPs, as well as T26, C27^+^, T28, and T30 in the HBPs. The relative peak intensity ($I_{\left(t\right)}/I_{0}$) of each imino proton, obtained from WMT experiments, was plotted as a function of delay time (Fig. 4A). The $k_{\mathrm{ex},1H}$ values were then extracted by fitting these data to Eqn 15, with the results summarized in Fig. 4B and Tables 1, 2, 3.

### Lifetimes of the closed and open states for PT‐ODN base pairs in the absence and presence of crowders

**Fig. 5:**
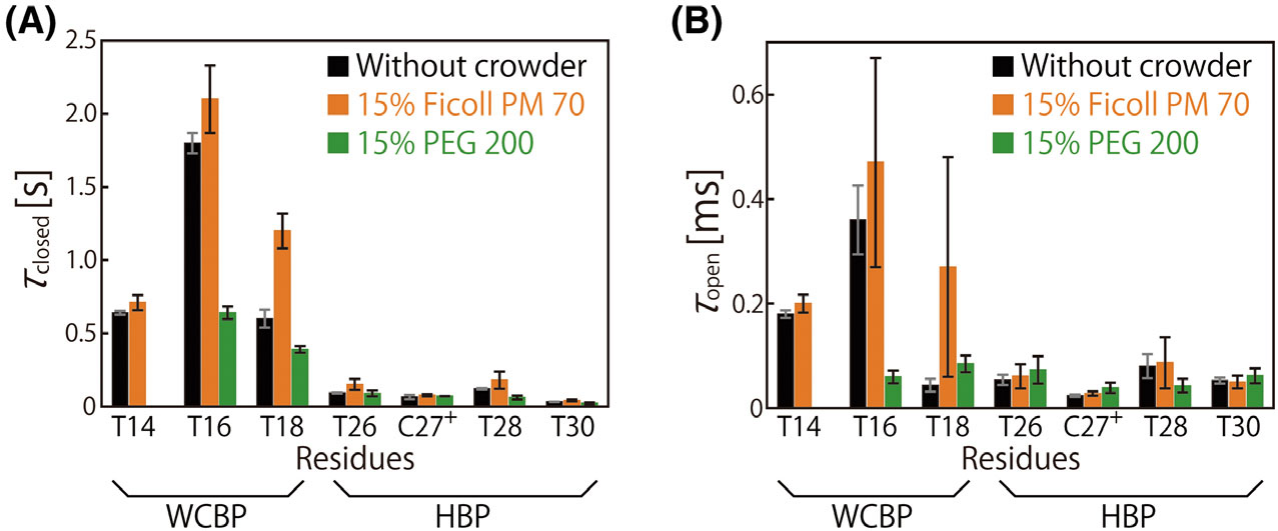
Lifetimes of the closed and open states for oligodeoxynucleotide that forms a parallel triplex structure (PT‐ODN). (A) $\tau_{\text{closed}}$ and (B) $\tau_{\text{open}}$ values determined for PT‐ODN. The lifetimes of the closed and open states ($\tau_{\text{closed}}$ and $\tau_{\text{open}}$) are defined as $1/k_{\text{open}}$ and $1/k_{\text{close}}$, respectively. The data are color‐coded based on experimental conditions: without crowder (black), with 15% Ficoll (orange), and with 15% PEG 200 (green). Under the PEG 200‐added condition, the T14 imino proton signal overlapped (Fig. 2C, top), preventing reliable peak picking and subsequent analysis. $\tau_{\text{closed}}$ and $\tau_{\text{open}}$ values were calculated from parameters obtained from one relaxation‐dispersion (RD) dataset and one water‐magnetization‐transfer (WMT) dataset, each acquired once per condition (*n* = 1 per condition). Details of the calculations of $\tau_{\text{closed}}$ and $\tau_{\text{open}}$ values are provided in the Results section under “Theoretical framework for analyzing base‐pair opening/closing (BPOC) dynamics” and “Lifetimes of the closed and open states for PT‐ODN base pairs in the absence and presence of crowders.” The uncertainties for $\tau_{\text{closed}}$ and $\tau_{\text{open}}$ values were calculated by error propagation from the Monte Carlo‐derived uncertainties of $k_{\mathrm{ex}}$ and $k_{\mathrm{ex},1H}$.

Values of $k_{\mathrm{ex}}$ and $k_{\mathrm{ex},1H}$ obtained from RD and WMT measurements, respectively, along with $k_{\mathrm{ex},\text{open}}$ values calculated using literature data (p*K*_a_, $k_{\text{coll}}$, and $k_{\mathrm{ex},\text{open}}^{\mathrm{int}}$) and experimental parameters ([HPO_4_^2−^] and pH) (see Previous Sections, Theoretical framework for analyzing base‐pair opening/closing (BPOC) dynamics and Calculating the exchange rate between imino and water protons in the open state ($k_{\mathrm{ex},\text{open}}$)), were substituted into Eqns 2,14. This enabled us to determine $k_{\text{open}}$ and $k_{\text{close}}$ values for the DNA triplex structure under MCE for the first time, without the need to adjust buffer conditions (summarized in Tables 1, 2, 3). The lifetimes of the closed and open states ($\tau_{\text{closed}}=1/k_{\text{open}}$ and $\tau_{\text{open}}=1/k_{\text{close}}$) were then calculated, with the results presented in Fig. 5A,B, and Tables 1, 2, 3. Standard errors for $\tau_{\text{closed}}$, $\tau_{\text{open}}$, and ${\Delta G}_{\text{open}}^{○}$ (discussed in the next section) were obtained from the standard errors of $k_{\mathrm{ex}}$ and $k_{\mathrm{ex},1H}$ using standard error‐propagation formulas.

In our study, the $\tau_{\text{closed}}$ values at 25 °C in the absence of crowders were generally on the millisecond timescale, ranging from 0.031 s to 0.64 s, with the exception of T16, which exhibited a $\tau_{\text{closed}}$ of 1.8 s (Table 1). Cain and Glick previously characterized base‐pair lifetimes in a DNA triplex (31‐mer) using conventional WMT experiments with various base catalysts present but in the absence of crowders [62], and they also reported millisecond‐scale lifetimes at 21 °C [62]. Therefore, the millisecond‐scale lifetimes we observed for base‐pairs in the triplex, determined from RD and WMT experiments together with literature‐derived $k_{\mathrm{ex},\text{open}}$ values, are consistent with these earlier findings.

The WCBPs exhibited longer closed‐state lifetimes ($\tau_{\text{closed}}$) than the HBPs under both dilute and MCEs. The addition of Ficoll PM 70 increased $\tau_{\text{closed}}$ values for T14, T16, and T18 in the WCBPs, as well as T26, C27^+^, T28, and T30 in the HBPs (Fig. 5A), indicating that Ficoll PM 70 extends the closed state lifetimes of all WCBPs and HBPs. In contrast, PEG 200 increased the $\tau_{\text{closed}}$ value only for C27^+^ in the HBPs, while it decreased $\tau_{\text{closed}}$ for the other residues (T16 and T18 in the WCBPs, and T26, T28, and T30 in the HBPs). Thus, PEG 200 generally shortens the closed‐state lifetimes of WCBPs and HBPs, except for C27^+^.

With the exception of T18, the WCBPs also generally exhibited longer open‐state lifetimes ($\tau_{\text{open}}$) than the HBPs under dilute conditions. The addition of Ficoll PM 70 increased $\tau_{\text{open}}$ values for T14, T16, and T18 in the WCBPs, as well as for T26, C27^+^, and T28 in the HBPs; however, T30 in the HBPs exhibited a lower $\tau_{\text{open}}$ value. Therefore, Ficoll PM 70 extends the open‐state lifetimes for all WCBPs and HBPs, with the exception of T30 in the HBPs. In contrast, PEG 200 increased $\tau_{\text{open}}$ values for T18 in the WCBPs and for T26, C27^+^, and T30 in the HBPs, while it decreased $\tau_{\text{open}}$ for T16 in the WCBPs and T28 in the HBPs. Thus, PEG 200 extends the open‐state lifetimes for most WCBPs and HBPs, except for T16 in the WCBPs and T28 in the HBPs.

Residues T14, T16, T18, T26, T28, and T30 are part of base triples located near the 3′ end (T14 [WCBPs], T30 [HBPs]), the 5′ end (T18 [WCBPs], T26 [HBPs]), or the central region (T16 [WCBPs], T28 [HBPs]) of the strand. Notably, T16 and T28, which uniquely showed a decrease in $\tau_{\text{open}}$ upon addition of PEG 200, are positioned in the central region of the strand.

Under dilute conditions, both the closed‐ and open‐state lifetimes were longer for residues belonging to the central base triple than for those near the 5′ or 3′ ends. This trend was also maintained in the Ficoll PM 70 MCE, suggesting that the central base triple is more resistant to transitions between states. In contrast, this trend was not observed under the PEG 200‐MCE, where the closed‐state lifetime for T26 (HBPs), located near the 5′ end, was longer than that for T28 (HBPs), located in the middle. Furthermore, T26 (HBPs) and T30 (HBPs), located near the 5′ and 3′ ends respectively, exhibited longer open‐state lifetimes than T28 (HBPs), located in the middle.

### ${\Delta G}_{\text{open}}^{○}$ values for PT‐ODN base pairs in the absence and presence of crowders

**Fig. 6:**
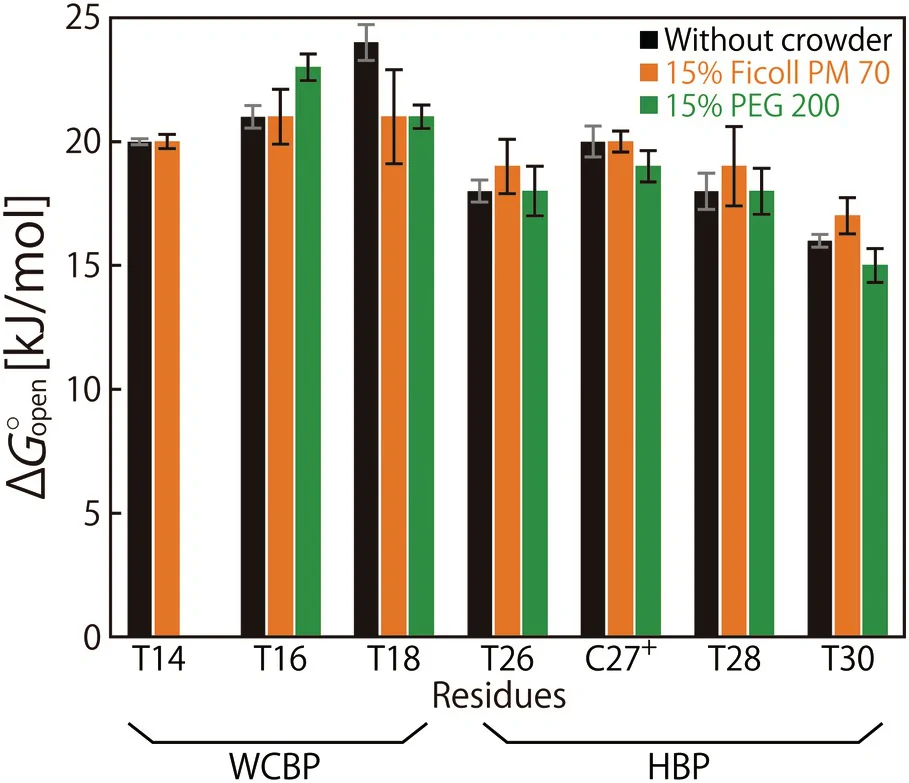
${\Delta G}_{\text{open}}^{○}$ values for oligodeoxynucleotide that forms a parallel triplex structure (PT‐ODN). The standard Gibbs energy difference between the closed and open states of the base pairs (${\Delta G}_{\text{open}}^{○}$) are defined as $-\mathrm{RT}\ln K_{\text{open}}$, $K_{\text{open}}=\frac{k_{\text{open}}}{k_{\text{close}}}=\frac{\tau_{\text{open}}}{\tau_{\text{closed}}}$. The data are color‐coded based on experimental conditions: without crowder (black), with 15% Ficoll (orange), and with 15% PEG 200 (green). Under the PEG 200‐added condition, the T14 imino proton signal overlapped (Fig. 2C, top), preventing reliable peak picking and subsequent analysis. ${\Delta G}_{\text{open}}^{○}$ values were calculated from parameters obtained from one relaxation‐dispersion (RD) dataset and one water‐magnetization‐transfer (WMT) dataset, each acquired once per condition (*n* = 1 per condition). Details of the calculations of ${\Delta G}_{\text{open}}^{○}$ values are provided in the Results section under “Theoretical framework for analyzing base‐pair opening/closing (BPOC) dynamics” and “Lifetimes of the closed and open states for PT‐ODN base pairs in the absence and presence of crowders.” The uncertainties for ${\Delta G}_{\text{open}}^{○}$ values were calculated by error‐propagation from the Monte Carlo‐derived uncertainties of $k_{\mathrm{ex}}$ and $k_{\mathrm{ex},1H}$ using.

Based on the $k_{\text{open}}$ and $k_{\text{close}}$ values, we next calculated the changes in standard Gibbs energy difference between the closed and open states (${\Delta G}_{\text{open}}^{○}$), defined as $G_{\text{open}}^{○}-G_{\text{closed}}^{○}=-\mathrm{RT}\;\ln k_{\text{open}},k_{\text{open}}=\frac{k_{\text{open}}}{k_{\text{close}}}=\frac{\tau_{\text{open}}}{\tau_{\text{closed}}}$. These ${\Delta G}_{\text{open}}^{○}$ values were determined for PT‐ODN both in the absence and in the presence of crowders, as summarized in Fig. 6 and Tables 1, 2, 3.

In the absence of a crowders, the ${\Delta G}_{\text{open}}^{○}$ values followed the order: $T18\;\left(\text{WCBPs}\right)>T16\;\left(\text{WCBPs}\right)>T14\;\left(\text{WCBPs}\right)=C27^{+}\;\left(\text{HBPs}\right)>T26\;\left(\text{HBPs}\right)=T28\left(\text{HBPs}\right)>T30\;\left(\text{HBPs}\right)$. A higher ${\Delta G}_{\text{open}}^{○}$value indicates greater stability of the closed state relative to the open state. The HBPs generally exhibited smaller ${\Delta G}_{\text{open}}^{○}$ values compared to the WCBPs, suggesting that HBPs are more prone to adopting the open state. This trend persisted even in the presence of Ficoll PM 70 or PEG 200, further supporting the conclusion that HBPs are more likely to reside in the open state than the WCBPs, regardless of crowding conditions. This observation is consistent with a previous study on the imino proton‐exchange process of PT‐ODN under dilute conditions [57].

Compared to the results obtained in the absence of a crowder, T18 exhibited a lower ${\Delta G}_{\text{open}}^{○}$ value in the presence of Ficoll PM 70, while T26, T28, and T30 showed higher values; T14, T16, and C27^+^ exhibited no significant changes. Focusing on the HBPs, $\tau_{\text{closed}}$ and $\tau_{\text{open}}$ increased for T26 and T28, with a more‐pronounced increase observed in $\tau_{\text{closed}}$, resulting in an overall increase in ${\Delta G}_{\text{open}}^{○}$. For T30, the closed‐state lifetime increased and the open‐state lifetime decreased, also leading to a higher ${\Delta G}_{\text{open}}^{○}$ value.

In contrast, upon addition of PEG 200, the ${\Delta G}_{\text{open}}^{○}$ values of T18, C27^+^, and T30 decreased, whereas the values for T16 increased; T26 and T28 showed no significant changes. For the HBP residue C27^+^, both $\tau_{\text{closed}}$ and $\tau_{\text{open}}$ increased, with a more pronounced increase in $\tau_{\text{open}}$, resulting in an overall decrease in ${\Delta G}_{\text{open}}^{○}$. For T30, the closed‐state lifetime decreased while the open‐state lifetime increased, leading to a decrease in ${\Delta G}_{\text{open}}^{○}$ value.

### The thermal stability of the triplex structure

**Fig. 7:**
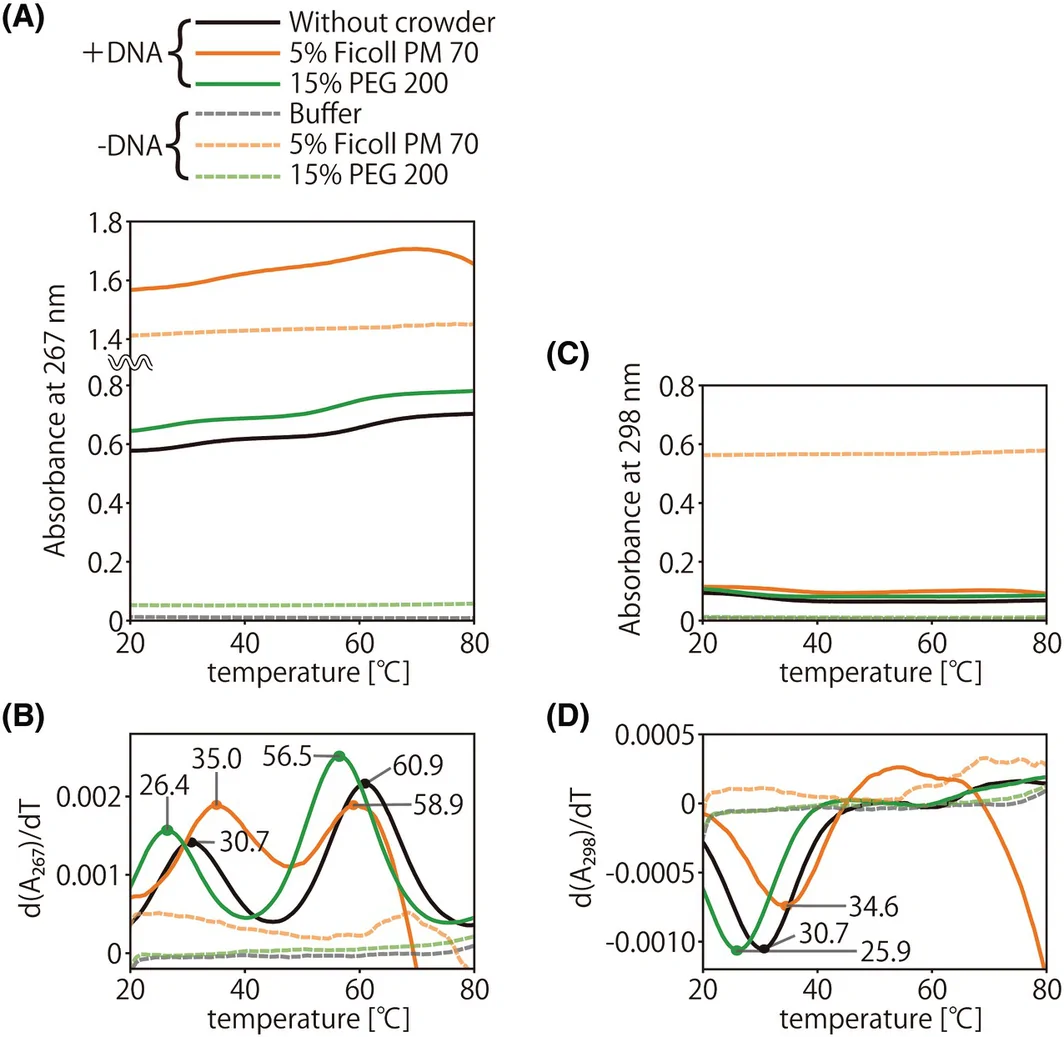
UV melting‐temperature analyses. (A) UV absorbance melting curves of oligodeoxynucleotide that forms a parallel triplex structure (PT‐ODN) at 267 nm, reporting overall thermal stability. The black curve corresponds to PT‐ODN under dilute conditions, whereas the orange and green curves correspond to PT‐ODN in the presence of 5% Ficoll PM 70 and 15% PEG 200, respectively. Dashed lines indicate control experiments performed without PT‐ODN to establish baseline absorbance. (B) First‐derivative plots of the curves shown in panel (A), used to determine melting temperatures ($T_{m}$). (C) UV absorbance melting curves of PT‐ODN at 298 nm, which report the stability of HBPs [49, 63]. (D) First‐derivative plots of the curves in panel (C), used to determine $T_{m}$ at 298 nm. Data were acquired once per condition (*n* = 1 per condition).

We investigated the effect of each crowder on the thermal stability of PT‐ODN using UV melting curves (Fig. 7A,C). The UV melting curve acquired at 267 nm in the absence of crowder displayed two transitions with $T_{m}$ values of 60.9 and 30.7 °C (Fig. 7B), whereas a single transition at $T_{m}$ = 30.7 °C was observed at 298 nm (Fig. 7D). Notably, a singular sigmoidal transition at 298 nm is characteristic of HBP‐containing DNA structures [49, 63]. We assigned the $T_{m}$ values of 30.7 and 60.9 °C to the triplex‐to‐duplex transition ($T_{m}^{T\to D}$ at 298 nm) and the duplex‐to‐single‐strand transition ($T_{m}^{D\to S}$ at 267 nm), respectively. Similar transition patterns were observed following the addition of either 5% Ficoll PM 70 or 15% PEG 200 (Fig. 7), with the corresponding $T_{m}$ values summarized in Table 7.

**Table 7:** $T_{m}$ values of PT‐ODN in the presence and absence of crowders.

		267 nm	298 nm
Without crowder	$T_{m}^{T\to D}$	30.7	30.7
	$T_{m}^{D\to S}$	60.9	‐
5% Ficoll PM 70	$T_{m}^{T\to D}$	35.0	34.6
	$T_{m}^{D\to S}$	58.9	‐
15% PEG 200	$T_{m}^{T\to D}$	26.4	25.9
	$T_{m}^{D\to S}$	56.5	‐

## Discussion

**Fig. 8:**
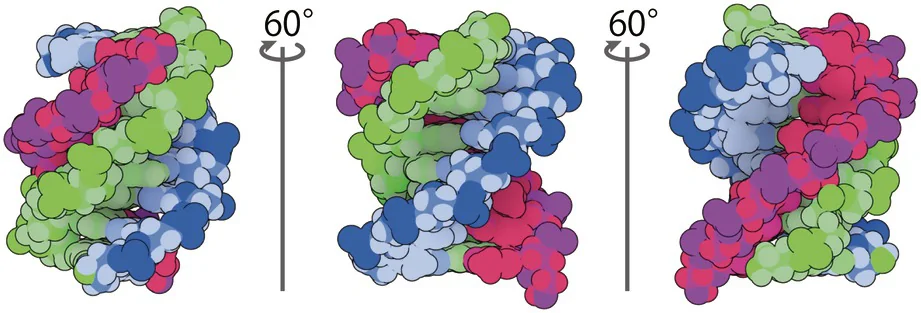
NMR structure of a DNA triplex with a sequence similar to that of oligodeoxynucleotide that forms a parallel triplex structure (PT‐ODN) (PDB: 1D3X) [65]. The green strand is polypurine; the blue strand is polypyrimidine and forms Watson–Crick base pairs (WCBPs); and the red strand is polypyrimidine and forms Hoogsteen base pairs (HBPs). The three panels show the structure from different orientations. The structure was rendered using Protein Imager [86] in stick representation with chains colored individually.

The RD method employed in this study is capable of detecting not only base‐pair opening and closing but also WCBP‐to‐HGBP (WC‐HG) exchange dynamics. In duplex DNA, WC‐HG exchange occurs when the purine glycosidic bond rotates from the anti to the syn conformation, disrupting the WC hydrogen bond (e.g., N1‐N3) and forming an HG bond (e.g., N7‐N3) with the same pyrimidine base. In a DNA triplex, the central purine in each base triple forms hydrogen bonds with two pyrimidine bases of each base triple, one via a WC interaction and the other via an HG interaction (Fig. 2A,B). Consequently, the anti‐to‐syn rotation of the purine required for WC‐HG exchange would necessitate the simultaneous disruption of both hydrogen bonds. These bonds must then be re‐formed in interchanged configurations (i.e., the WC and HG base‐pairing partners swapped). This is not an easy process. Furthermore, the space for purines to rotate in the crowded triplex DNA structure is not enough (Fig. 8). As a result, WC‐HG exchange in triplex DNA is expected to occur at a significantly slower rate than in duplex DNA.

In duplex DNA, the exchange rate constant ($k_{\mathrm{ex}}$) for WC‐HG exchange, as measured by RD experiments, is typically on the order of 10^3^ s^−1^ [14, 15, 16, 17, 64]. As discussed above, $k_{\mathrm{ex}}$ in the triplex DNA is expected to be substantially lower than that in the duplex DNA. However, the $k_{\mathrm{ex}}$ values observed in this study ranged from 10^3^ to 10^4^ s^−1^, comparable to or even exceeding those for WC‐HG exchange in duplex DNA. These unexpectedly high $k_{\mathrm{ex}}$ suggest that the exchange process detected by RD in our system predominantly reflects base‐pair opening and closing dynamics rather than WC‐HG exchange.

Higher ${\Delta G}_{\text{open}}^{○}$ values were observed for WCBPs than for HBPs both in the absence and in the presence of crowders (Fig. 6). This behavior likely reflects the structural consequences of base pair opening within the DNA triplex. The third strand binds in the major‐groove and contribute additively to triplex formation, without substantially altering the helical parameters of the underlying B‐form DNA duplex [65]. In contrast, opening of a WCBP disrupts local duplex stability, and this destabilization can propagate to nearby regions, including adjacent HBPs within the same or neighboring triplex planes. As a result, transitions from the closed to open state are thermodynamically less favorable for WCBPs than for HGBPs, reflected in their high ${\Delta G}_{\text{open}}^{○}$ values.

The addition of Ficoll PM 70 increased $T_{m}^{T\to D}$ by 3.9 °C (Table 7), indicating enhanced thermal stability of the triplex structure, likely due to a stronger excluded‐volume effect. In contrast, the addition of PEG 200 decreased $T_{m}^{T\to D}$ by 4.8 °C (Table 7), suggesting reduced thermal stability attributable to lower water activity. The contrasting effects of Ficoll PM 70 and PEG 200 on the thermal stability of the PT‐ODN triplex structure underscore the complex interplay between excluded‐volume effects and water activity [45, 46].

The enhanced thermal stability of the triplex structure in the presence of Ficoll PM 70 indicates that the transition to the duplex structure is more strongly inhibited than in its absence. This interpretation is supported by the observed changes in ${\Delta G}_{\text{open}}^{○}$ (Fig. 6), which increased for three residues (T26, T28, and T30), decrease for one residue (T18), and remained unchanged for three residues (T14, T16, and C27^+^). Since $T_{m}^{T\to D}$ corresponds to the triplex‐to‐duplex structural transition, the overall increase in thermal stability observed in the UV melting experiments can be attributed to the cumulative effect of the higher ${\Delta G}_{\text{open}}^{○}$ values for the T26, T28, and T30 HBP residues.

In contrast, the addition of PEG 200 decreased the thermal stability of the triplex structure, thereby promoting its transition to the duplex form. This reduction in thermal stability corresponds to the observed changes in ${\Delta G}_{\text{open}}^{○}$ (Fig. 6), which decreased for three residues (T18, C27^+^, T30), showed no significant change for two residues (T26 and T28), and increased for one residue (T16). Thus, the overall decrease in thermal stability can be attributed to the cumulative effect of the lower ${\Delta G}_{\text{open}}^{○}$ values for the C27^+^ and T30 HBP residues.

Although MCE does not alter the overall structure of the DNA triplex, it influences the lifetimes of the closed and open states, as well as the standard Gibbs energy differences between these states. Ficoll PM 70 and PEG 200 exert distinct MCE effects on the BPOC dynamics of the triplex; the former primarily acts through the excluded‐volume effect, whereas the latter mainly affects water activity. Our findings suggest that the MCE, such as that in living cells, can significantly impact the BPOC dynamics of base pairs within DNA triplex structures.

In a biological context, the MCE fluctuates through various mechanisms. For example, activation of the mTORC1 signaling pathway increases the intracellular ribosome concentrations [66], thereby enhancing the excluded‐volume effect and potentially decreasing water activity. Additionally, changes in cell volume during the cell cycle [67] or senescence [68] alter intracellular protein concentrations, influencing both the excluded‐volume effect and water activity. Similar changes can occur during osmotic stress, where water efflux and a reduction in cell volume [69, 70, 71] increase crowding. Cells also accumulate osmolytes, such as sorbitol or betaine, to adapt to osmotic stress [70, 71], further decreasing water activity. Our findings suggest that DNA triplexes may respond to MCE fluctuations induced by cellular processes, potentially influencing gene expression, replication, and genetic instability.

Furthermore, the addition of PEG 200 suggests the possibility of residue‐specific effects within MCE, as the open‐state lifetimes in the triplex decreased near the center and increased near the ends (Fig. 5B). These residue‐specific changes in BPOC dynamics may lead to localized base‐pair openings that create specific binding sites for DNA‐binding proteins, raising the intriguing possibility that some DNA triplex–protein interactions may occur exclusively under MCEs.

These findings also raise the interesting possibility that molecular crowding‐dependent alterations in BPOC dynamics of triplex structures may affect how proteins or enzymes that recognize triplex‐forming regions interact with such structures, an area that warrants future investigation.

## Material and methods

### Oligonucleotides

HPLC‐grade PT‐ODN was purchased from FASMAC and dissolved in 20 mm sodium phosphate buffer (pH 7.0) containing 8 mm NaH_2_PO_4_, 12 mm Na_2_HPO_4_, and 168 mm NaCl. The solution was heated at 95 °C for 5 min, then gradually cooled to 30 °C to facilitate annealing.

### NMR spectroscopy

All NMR samples were prepared using 1.5 mm PT‐ODN in the abovementioned 20 mm sodium phosphate buffer (pH 7.0) containing 5% ^2^H_2_O (D_2_O). Crowder‐containing samples were prepared by adding 15% (w/v) of either Ficoll PM 70 (GE Healthcare) or PEG 200 (Nacalai Tesque). NMR data were acquired using Bruker AVANCE III HD 600 and 800 spectrometers, both of which were equipped with ultra‐high‐sensitivity cryoprobes and Z‐gradients. All experiments were conducted at 25 °C.

### On‐resonance ^1^H $R_{1\rho }$ relaxation dispersion (RD) experiments

RD experiments were performed using a previously reported pulse sequence [20, 72]. The carrier frequency was set on‐resonance for each imino proton resonance. NMR spectra were processed using a custom Python program that integrates nmrglue [73] for spectral processing, lmfit [74] for fitting, and SymPy [75], SciPy [76], and NumPy [77] for numerical calculations, with Matplotlib [78] used to represent the data graphically.

The effective $R_{1\rho }$ relaxation rate was calculated using Eqn 8: [20].

(8) $$R_{1\rho }=-\frac{1}{T}\ln\left(\frac{I_{\mathrm{SL}}}{I_{0}}\right),$$

where $I_{\mathrm{SL}}$ and $I_{0}$ are signal intensities acquired with and without spin‐lock, respectively. ^1^H $R_{1\rho }$ RD spectra were recorded at spin‐lock powers ($\omega_{1}/2\pi$) in the 0.5–15 kHz range, with a constant relaxation period T of 20 ms.

RD profiles were fitted to a two‐state exchange model (A⇄B) as described by Eqn 9: [20, 21].

(9) $$R_{1\rho }=R_{2}^{0}+\frac{p_{A}p_{B}\Delta \omega^{2}k_{\mathrm{ex}}}{\frac{\omega_{\mathrm{Ae}}^{2}\omega_{\mathrm{Be}}^{2}}{\omega_{e}^{2}}+k_{\mathrm{ex}}^{2}-p_{A}p_{B}\Delta \omega^{2}\left(1+\frac{2k_{\mathrm{ex}}^{2}\left(p_{A}\omega_{\mathrm{Ae}}^{2}+p_{B}\omega_{\mathrm{Be}}^{2}\right)}{\omega_{\mathrm{Ae}}^{2}\omega_{\mathrm{Be}}^{2}+\omega_{e}^{2}k_{\mathrm{ex}}^{2}}\right)},$$

where:

(10) $$\omega_{\mathrm{Ae}}^{2}=\omega_{1}^{2},$$

(11) $$\omega_{\mathrm{Be}}^{2}=\Delta \omega^{2}+\omega_{1}^{2},$$

(12) $$\omega_{e}^{2}=\Omega_{A}^{2}+\omega_{1}^{2},$$

(13) $$\Omega_{A}=-\frac{p_{B}k_{\mathrm{ex}}^{2}\Delta \omega }{k_{\mathrm{ex}}^{2}+\Delta \omega^{2}},$$

(14) $$k_{\mathrm{ex}}=k_{\text{close}}+k_{\text{open}}.$$

Here, $R_{2}^{0}$ is the intrinsic transverse relaxation rate, $p_{A}$ and $p_{B}$ (with $p_{A}+p_{B}=1$) are the populations of the major and minor states, respectively, $k_{\mathrm{ex}}$ is the conformational exchange rate, and Δω is the chemical shift difference between the two states. The effective resonance frequencies of the major and minor states ($\omega_{\mathrm{Ae}}$ and $\omega_{\mathrm{Be}}$) and the average effective field frequency ($\omega_{e}$) are defined by Eqns (10), (11), (12), respectively, while the average resonance offset ($\Omega_{A}$) is defined by Eqn 13. Errors were determined from 2000 Monte Carlo simulations of synthetic intensity datasets constructed using the noise levels present in the experimental spectra [79]. Each synthetic dataset was independently fitted, and the resulting distribution of fitted parameters was used to estimate standard errors.

### Water‐magnetization‐transfer (WMT) experiments

WMT experiments were performed using a selective 180° pulse (Q3 pulse) to invert the water protons, after which variable delays $\left(t\right)$were applied [53, 59, 80, 81]. Imino proton signals were subsequently acquired using the SOFAST technique [82, 83], with PC9 and rSNOB pulses used to excite and invert. WMT spectra were processed using the custom Python program described above. The water–imino proton‐exchange rate $k_{\mathrm{ex},1H}$ was quantified by fitting the relative peak intensities $I_{\left(t\right)}/I_{0}$ of the imino protons to Eqn 15:

(15) $$I_{\left(t\right)}/I_{0}=1-2\frac{k_{\mathrm{ex},1H}}{R_{1w}-R_{1a}}\left(e^{-R_{1a}t}-e^{-R_{1w}t}\right),$$

where $I_{0}$ and $I_{\left(t\right)}$ are the signal intensities of the imino proton at the initial delay time (zero) and at delay time t, respectively [59]. The longitudinal relaxation rate constants for the imino protons ($R_{1a}$) and water protons ($R_{1w}$) were obtained by subjecting the imino protons to inversion‐recovery experiments [59] and the water protons to saturation‐recovery experiments [37]. Errors were determined from 500 Monte Carlo simulations of synthetic intensity datasets constructed using the noise levels present in the experimental spectra [37]. Each synthetic dataset was independently fitted, and the resulting distribution of fitted parameters was used to estimate standard errors.

### UV melting‐temperature analyses

UV melting‐temperature experiments were performed using a JASCO V‐750 UV–VIS spectrophotometer. Absorbance was monitored at 267 and 298 nm as the temperature gradually decreased from 95 to 0 °C at 0.5 °C·min^−1^. A 1.5 μm solution of the PT‐ODN in sodium phosphate buffer (pH 7.0) was prepared, with either Ficoll PM 70 or PEG 200 added to final concentrations of 5% or 15%, respectively, to simulate MCEs. Due to Ficoll PM 70's inherent UV absorption and scattering, conducting experiments with more than 5% of this agent was challenging. $T_{m}$ values were determined from the peaks in the first‐derivative absorbance versus temperature curves, which correspond to melting‐transition inflection points.

## Author contributions

TS, YY, TN, and MK designed the experiments. TS, YY, and TN conducted the experiments and analyzed the data. TS, YY, TN, and MK interpreted the results and wrote the manuscript.

## Conflict of interest

The authors declare no conflict of interest.

## Data Availability

The data that support the findings of this study are available from the corresponding authors [katahira.masato.6u@kyoto-u.ac.jp and nagata.takashi.6w@kyoto-u.ac.jp] upon reasonable request.
